# Multiple faces elicit augmented neural activity

**DOI:** 10.3389/fnhum.2013.00282

**Published:** 2013-06-14

**Authors:** Aina Puce, Marie E. McNeely, Michael E. Berrebi, James C. Thompson, Jillian Hardee, Julie Brefczynski-Lewis

**Affiliations:** ^1^Department of Radiology, Center for Advanced Imaging, School of Medicine, West Virginia UniversityMorgantown, WV, USA; ^2^Department of Radiology, School of Medicine, West Virginia UniversityMorgantown, WV, USA; ^3^Department of Neurobiology and Anatomy, School of Medicine, West Virginia UniversityMorgantown, WV, USA; ^4^Department of Psychological and Brain Sciences, Indiana UniversityBloomington, IN, USA; ^5^Centene CorporationSt. Louis, MO, USA; ^6^Department of Sport Psychology, College of Physical Activity and Sport Sciences, West Virginia UniversityMorgantown, WV, USA; ^7^Department of Psychology, George Mason UniversityFairfax, VA, USA; ^8^Department of Psychiatry, University of MichiganAnn Arbor, MI, USA

**Keywords:** faces, ERPs, P100, N170, P250, multiple faces, brightness, contrast

## Abstract

How do our brains respond when we are being watched by a group of people?Despite the large volume of literature devoted to face processing, this question has received very little attention. Here we measured the effects on the face-sensitive N170 and other ERPs to viewing displays of one, two and three faces in two experiments. In Experiment 1, *overall* image brightness and contrast were adjusted to be constant, whereas in Experiment 2 local contrast and brightness of individual faces were not manipulated. A robust positive-negative-positive (P100-N170-P250) ERP complex and an additional late positive ERP, the P400, were elicited to all stimulus types. As the number of faces in the display increased, N170 amplitude increased for both stimulus sets, and latency increased in Experiment 2. P100 latency and P250 amplitude were affected by changes in overall brightness and contrast, but not by the number of faces in the display *per se*. In Experiment 1 when overall brightness and contrast were adjusted to be constant, later ERP (P250 and P400) latencies showed differences as a function of hemisphere. Hence, our data indicate that N170 increases its magnitude when multiple faces are seen, apparently impervious to basic low-level stimulus features including stimulus size. Outstanding questions remain regarding category-sensitive neural activity that is elicited to viewing multiple items of stimulus categories other than faces.

## Introduction

Why do we feel self-conscious when we are being watched by a group of people? If we are required to perform a certain task, then the fact that we know that we are being watched by others can potentially alter our behavior (Conty et al., 2010a,b). It is the scrutiny of others and their gaze falling on us that is said to be at the heart of the problem. Perceived direct gaze from others is thought to increase arousal in the individual being watched (Conty et al., 2010a,b). Interestingly, this effect can be seen even when isolated eyes, devoid of the rest of the face, are presented (Conty et al., 2010b). What happens at the neural level when we feel we are being watched by more than one person? Despite this situation occurring on a daily basis, it has nevertheless received little scrutiny, despite the extremely large literature on the neural correlates of various types of anxiety. Social anxiety has typically been investigated in terms of the brain's response to viewing a face with an emotional expression such as fear or anger, with personality type influencing how the brain responds to the facial expression (Cremers et al., 2010; Demenescu et al., 2010; Calder et al., 2011).

There is now a very large neuroimaging literature examining neural responses to static faces and objects, which has identified a number of active loci in ventral and lateral occipitotemporal cortex centered on the fusiform gyrus, inferior occipital gyrus and superior temporal sulcus in functional magnetic resonance imaging (fMRI) studies (Haxby et al., 2000; Tsao and Livingstone, 2008; Towler and Eimer, 2012). Neurophysiologically, images of static faces and objects elicit a series of event-related potential (ERP) components, one of which is the N170 in electroencephalography (EEG) or the M170 in magnetoencephalography (MEG). The majority of studies have reported that N170 tends to be larger to face stimuli relative to other stimulus categories including objects, and tends to have larger amplitude and greater spatial extent in the right occipitotemporal scalp (Bentin et al., 1996; Towler and Eimer, 2012). These findings have prompted the idea that N/M170 is sensitive to the categorical nature of the stimulus, and there is an interesting debate in the literature as to its functional significance e.g., (Rossion et al., 2003; Meeren et al., 2008) that is beyond the scope of the current manuscript. P100, an ERP component that precedes N170, also can exhibit larger amplitudes to faces relative to other stimulus categories in children and adults alike, which are proposed to be driven more by low-level visual cues in the stimulus (Taylor et al., 2004; Kuefner et al., 2010; Rossion and Caharel, 2011).

With respect to the N170 elicited to the face, it has been proposed that it is the eyes that drive most of the N170 response when a face stimulus is viewed, and this has been based largely on the observation that eyes in isolation produce N170s that are significantly larger and later than those seen to the full face (Itier et al., 2006; Itier and Batty, 2009). Multiple studies have shown robust ERP activity to viewing a single face averting its gaze or gazing directly at the viewer (Puce et al., 2000, 2003; Conty et al., 2007; Itier et al., 2007; George and Conty, 2008; Itier and Batty, 2009). The posterior temporal N170 or M170 changes its amplitude as a function of gaze direction, which might be modulated by changes in social attention. In a linear array of three faces the initial stimulus in the trial consists of a central face with direct gaze (at the viewer) and two flankers with averted gaze in the same direction. Then after a period of time the central face averts its gaze and the flanker faces do not change their (already) deviated gaze. N170 ERPs are elicited to the gaze change by the central face in all stimulus conditions (where social context has been varied as a function of direction of averted gaze in the central face). Interestingly, N170 amplitudes and latencies are unaffected by the social context of the gaze aversion, unlike subsequent ERP components at around 350–500 ms which differentiated according to social context (Carrick et al., 2007). However, the number of faces being viewed in each trial was always kept constant—a very unrealistic situation to what is encountered on a daily basis, where we interact with individuals as they come and go in groups or in isolation.

A potential problem that is created in varying the number of faces or individuals in the display lies in the changes that can be induced in the overall luminance, contrast and spatial frequency of the image. Similarly, changes in the visual scene or its content, such as material taken from cinematic movies, where visual stimulation is effectively uncontrolled have these same potential drawbacks. Yet, in order to really begin to understand the neural bases of interactions with our environment and with other individuals, it is necessary to use dynamic visual displays that vary their content and context. Remarkably, in fMRI studies similar activation patterns have been documented in populations of subjects to these uncontrolled visual stimuli relative to other previous (controlled) studies in the field e.g., (Bartels and Zeki, 2004; Hasson et al., 2004, 2010). In some cases, activation in additional brain regions was also demonstrated (Hasson et al., 2010). Studies of naturalistic visual stimulation of EEG/MEG are not numerous, but focal EEG changes (as determined by neural source modeling) have been demonstrated on millisecond time-scale relative to slower time-courses in fMRI using visual stimulation that consists of a cinematic movie (Whittingstall et al., 2010). Indeed, invasive EEG recordings in humans demonstrate that naturalistic (audio) visual stimulation elicits category-selective neural activity, which appears to be more selective than that reported for fMRI (Privman et al., 2007; Meshulam et al., 2013), and more temporally extensive relative to the presentation of static stimuli (Senkowski et al., 2007).

One way to explicitly study the effects of multiple stimulus items have on neural activity is by numerosity judgment. Numerosity can be judged on either a temporal or spatial scale. Spatial numerosity judgments can be made to multiple stimuli in homogeneous arrays (as in the current study), or in mixed arrays with multiple targets and distractors (Pagano and Mazza, 2012). For these mixed arrays of targets and distractors, the main ERP of interest has been the N2pc—a parietal scalp potential that is sensitive to the spatial position (lateralized of the target stimulus—which is modulated monotonically by target number for explicit judgments of numerosity (Pagano and Mazza, 2012; Mazza et al., 2013) or for judgments involving subitizing (Ester et al., 2012). The behavior of N2pc and N1 have been dissociated in ERP studies, where N1 has been found to be modulated by increasing item number when targets are presented without distracters, whereas N2pc will exhibit modulation as a function of numerosity in all types of displays (Mazza et al., 2013). Interestingly, the number of items to be processed will also produce temporally and spatially dissociable neural activity that distinguishes explicit counting from subitizing (Vuokko et al., 2013), although variables such as the visual cue size (when dealing with the spatial extent in dot displays) may greatly influence the ERP measures (Gebuis and Reynvoet, 2013).

Here we studied how neural responses, specifically P100, N170 (N1), and P250, varied when individuals face the direct gaze of *differing* numbers of faces—a likely scenario that would be often encountered in naturalistic stimulation. While we predicted that augmented neural responses would be elicited when multiple faces are viewed relative to a solitary face, however, we were uncertain if the increase in response magnitude would scale proportionally as a function of face number, or would be constant for numbers of faces greater than one. We performed two experiments to examine this question. In Experiment 1, overall brightness and contrast of the visual display were adjusted to be constant. In Experiment 2, we chose to use a display in which the overall luminance and contrast (with respect to the faces themselves) were not constant, so as to elicit a situation that might occur during a more naturalistic viewing situation such as when viewing movie-based materials where visually stimulus characteristics such as brightness, contrast, and spatial frequency are not controlled. Subjects made a forced 3-choice button press to indicate how many faces were presented in the display in which no distracter items were present.

## Materials and methods

Two experiments were performed, each on a different group of subjects. Below we describe the attributes of the participants, as well as data acquisition and analysis separately in each section.

### Participants

All studied participants had normal or corrected-to-normal vision. No participant reported a previous history of psychiatric or neurological illness. Both experiments were approved by the West Virginia University Institutional Review Board for the Protection of Human Research Subjects.

#### Experiment 1

Fourteen healthy volunteers participated in this study. Due to technical difficulties, data from one of the 14 participants were excluded. Hence, for the 13 participants included in the data analyses mean age was 22.1 ± 3.4 years, and there were eight females. Eleven participants were right-handed and two were left-handed.

#### Experiment 2

A total of 18 right-handed healthy volunteers were studied. Data from four participants in this group were excluded due to excessive artifacts such as blinking and EMG activity. Hence, for the final group of 14 participants mean age was 21.6 ± 3.3 years, and there were eight males and six females.

### Visual stimulation

For both experiments, participants were seated in a comfortable reclining chair in a quiet, dimly lit room. Each participant fixated on the center of a visual display where grayscale images of one, two, or three smiling faces were presented. Overall, the total number of male and female faces appearing in each stimulus condition were equated, as were the repeats of each face image. Stimuli were generated from a base set of 30 female and 30 male faces which had been photographed with identical lighting and angle. All faces gazed directly at the observer and displayed a happy expression. In Experiment 1, stimuli were adjusted to ensure that *overall* brightness and contrast of the visual display was constant. The adjustments were made based on measurements of grayscale luminance and contrast values in each image using Photoshop. The contrast value was effectively a root-mean-square calculation (across the entire image) and the brightness measure was the mean luminance across the entire image (including background for the stimulus). This meant that the local brightness and contrast of the individual faces in the image varied systematically due to the need to compensate overall brightness and contrast in the image. The rectangular image with the faces was presented on a gray computer screen. In Experiment 2, stimuli were presented without these adjustments, hence preserving the *local* brightness and contrast characteristics of the stimulus image across the experiment, despite having large variations in the overall brightness and contrast of the stimuli as a function of face number. The image itself was presented on a black computer screen. As the same stimulus faces were utilized in the two experiments, we studied two separate groups of participants, so that any effects due to familiarity or repetition would not act as cross-session confounds.

In both experiments, each stimulus remained on the screen for 1 s until replaced by a plain background screen for 1 s. (Experiment 1: 50% gray overall background with an irregular pattern inset; Experiment 2: white background). The three stimulus conditions (single face, two faces, three faces) were presented in pseudorandom order, with no consecutive images of a particular stimulus type. There were a total of 60 exemplars for each trial type. Stimuli were presented in two to three separate viewing runs of 3–5 min each. Subjects were asked to respond to each stimulus and indicate the number of faces in the display by pressing one of three response buttons.

#### Experiment 1 [preserved global brightness and contrast (GBC)]

**Figure 1:**
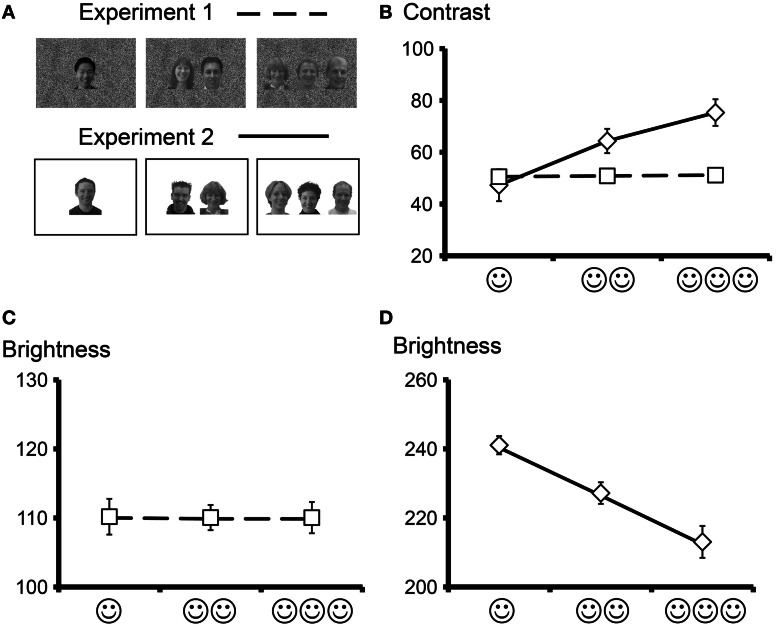
**Overall brightness and contrast (means and standard errors) for the two face stimulus sets. (A)** Sample stimuli from Experiment 1 (GBC stimulus set, top panel) and Experiment 2 (LBC stimulus set, bottom panel) showing one, two, and three faces. Grayscale faces appeared on a scrambled grayscale (Experiment 1) or a white (Experiment 2) background. **(B)** Overall contrast as a function of face number for Experiment 1 (broken line) and Experiment 2 (solid line). Average contrast increased as a near linear function of increasing number of faces in the visual display for Experiment 2 and was constant for Experiment 1. **(C)** Overall brightness was adjusted to remain constant regardless of the number of faces in the visual display for Experiment 1. **(D)** Overall brightness decreased linearly as a function of face number in Experiment 2.

Participants sat positioned approximately 195 cm from a white wall on which stimuli were presented on a gray background with a grayscale rectangle consisting of an irregular patterned background housing the face stimuli (see Figure 1A top panel) which subtended a total visual angle of 25.4 (horizontal) × 17.5 (vertical) degrees, respectively. Average visual angles subtended by images of one, two, and three faces were 8.9 × 26.0, 12.4 × 26.0, and 18.8 × 26.0 degrees, respectively. Overall mean contrast (Figure 1B, broken line) and brightness (Figure 1C) of the images were plotted as a function for each stimulus category and did not change as a function of increasing face number in the GBC stimulus set.

#### Experiment 2 [preserved local brightness and contrast (LBC)]

Participants sat positioned approximately 165 cm from a 33 cm computer monitor. Stimuli were presented on a black background with a white rectangle housing the face stimuli (see Figure 1A bottom panel) that subtended a total visual angle of 4.0 (horizontal) × 3.0 (vertical) degrees. Average visual angles subtended by images of one, two, and three faces were 0.7 × 1.3, 2.1 × 1.3, and 3.3 × 1.3 degrees, respectively. Mean brightness and contrast were measured for each image using Photoshop 7.1 (Adobe Systems Inc.) and the overall mean brightness and contrast plotted as a function for each stimulus category. There was a near linear increase in overall contrast (Figure 1B, solid line) and decrease in overall brightness (Figure 1D) as a function of increasing face number in the LBC stimulus set. Having said that, the face stimuli remained unaltered with respect to their local luminance and contrast, unlike in the GBC where the brightness and contrast of the faces had to be altered to ensure a constant overall image brightness and contrast.

### EEG recordings

For both experiments an identical recording procedure was used. Each participant was fitted with a 128 channels Neuroscan Electrocap with sintered Ag/AgCl electrodes. A dual reference electrode was placed on either side of the nose and a midline frontal ground electrode was located on the electrode cap. Electrodes were also placed to record horizontal electrooculogram (EOG) from the outer canthus of each eye and vertical EOG from above and below the left eye. All electrode impedances were below 15 kΩ. Continuous EEG and EOG were recorded from 128 channels on a Neuroscan Synamps amplifier using a band pass filter of 0.1–100 Hz and a gain of 5000 while each participant performed the task. Subjects were asked to keep movements to a minimum and, if possible, to restrict blinking to moments between stimulus presentations.

### EEG and ERP data analysis

For both experiments an identical data analysis procedure was utilized. The continuous EEG file was epoched, by segmenting the EEG record into 1020 ms epochs starting with a 100 ms pre-stimulus baseline. Trials with artifacts in the data were identified using a two-stage artifact detection procedure. First, an automated procedure identified and epochs rejected if activity exceeded ±75 μV, so as to reject epochs with eyeblinks and head/neck movements. Second, all remaining epochs were visually inspected to eliminate any other epochs containing additional artifacts. All trials with artifacts were excluded from further analysis.

The epoch baseline was adjusted by subtracting the mean amplitude of the pre-stimulus period from all data points within the epoch. EEG epochs were then averaged by stimulus type for each individual subject. Data were additionally digitally filtered using a 60 Hz band pass with a zero phase shift. Average ERP waveforms were generated for each subject and each condition for both experiments. Subject data were included in the final analysis if there were at least 45 trials per condition that could contribute to the average (of a total of 60 trials/condition). In Experiment 1 no subjects were excluded for this reason, and in Experiment 2 the data of 4 subjects were excluded due to this minimum trial number criterion.

For each experiment, grand average ERP waveforms were created for each condition across each subject group. Grand average ERP waveforms were visually inspected at time points corresponding to ERP components. For both experiments, the data of each individual participant were screened to identify ERP component peak latencies and amplitudes using a semi-automated procedure. A latency window specific to the ERP component of interest was created and a peak-picking algorithm identified the corresponding ERP peak. For P100 we chose a latency interval of 68–140 ms, and for the subsequent ERPs latency interval of 116–212 ms (N170), 170–290 ms (P250), and 312–650 ms (P400) were used. The peak amplitudes and latencies for each ERP component in an electrode cluster in each hemisphere (see below) were averaged and these data formed the input for the statistical analysis.

For both experiments topographic voltage maps were created at time points corresponding to ERP peaks so that the scalp distributions of the ERP activity could be visualized. Clusters of electrodes showing maximum ERP component amplitudes were identified in the bilateral temporo-occipital scalp, consistent with electrode positions that showed maxima in our previous studies (Puce et al., 2000, 2003; Brefczynski-Lewis et al., 2011), which were chosen to form an even cluster around electrodes P8, P08, and P10, and their respective left hemisphere homologues. These 10-10 electrode positions have been typically observed to produce N170s of maximal amplitude in previous studies. All results described in the subsequent sections are derived from data using these electrode clusters.

### Statistical analysis of ERP data

An identical statistical analysis was performed for each experiment. For each participant, ERP data were averaged among adjacent selected electrodes in areas where the greatest activity was observed in the topographic maps. Average ERP peak latencies and amplitudes were calculated for each four-electrode cluster located on the temporo-occipital scalp in each hemisphere for each subject. These data formed the input for the statistical analysis.

For each experiment, differences in ERP peak latency and amplitude as a function of condition were assessed by performing a General Linear Model analysis with factors of Face Number (One, Two, Three) × Hemisphere (Left, Right). Statistically significant results were identified as p < 0.05 (Greenhouse–Geiser corrected), which were then followed up with contrasts using SPSS V15 (Bonferroni corrected). Hemisphere here refers to the side of EEG recording.

## Results

### Behavior

In Experiment 1 subjects performed with a high-degree of accuracy, with accuracy rates being 98.6, 98.9, and 98.5% for 1, 2, and 3 faces, respectively. Reaction times for 1, 2, and 3 faces, respectively were 538.5 ± 68.5 ms, 541.6 ± 65.2 ms), and 541.2 ± 84.6 ms (mean ± standard deviation). Accuracy and response times did not differ as a function of condition, as shown by one-way ANOVA. Behavioral data from Experiment 2 were collected and analyzed, however, due to an issue with digital archiving could not be accessed.

### ERP data

**Figure 2:**
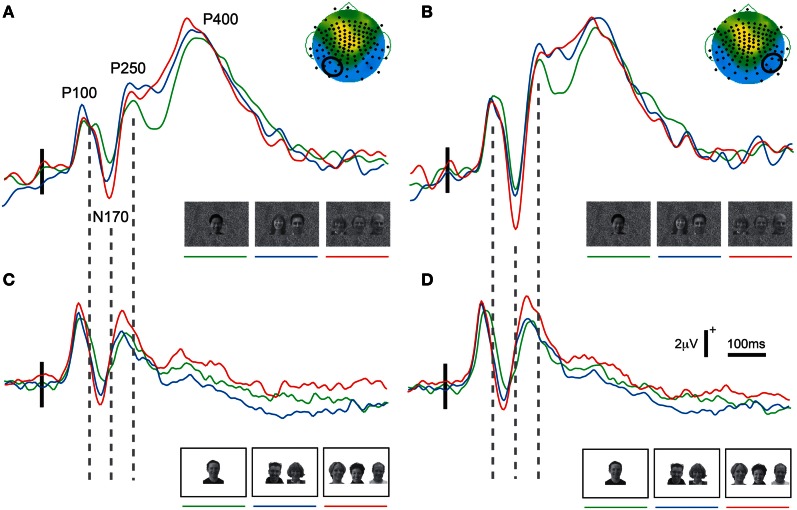
**Group average ERPs as a function of stimulus set and hemisphere.** Data show clear P100, N170, P250, and P400 activity in the right **(A,C)** and left **(B,D)** hemispheres for all stimulus conditions. Different ERP waveforms increasing number of faces in the display in all plots from green to blue to red. **(A,B)** Data from Experiment 1 (GBC stimulus set). In addition to the earlier ERP components, a prominent P400 is visible in both hemispheres for all stimulus conditions. **(C,D)** Data from Experiment 2 (LBC stimulus set). A clear P100, N170, and P250 is seen. The vertical broken lines between parts **(A)** and **(C)**, and between **(B)** and **(D)** demonstrate a clear latency shift for all ERP components across the two stimulus sets in both hemispheres, with shorter latencies occurring for stimuli with greatest overall brightness and contrast. Legend: horizontal and vertical calibration bars in **(C)** apply to all parts of the figure. Small vertical solid line overlying the earlier part of the ERP waveforms indicates stimulus delivery relative to a 100 ms pre-stimulus baseline. The small topographic maps (taken from the three face condition in Figure 3) in the top right corners of **(A,B)** highlight a four-electrode cluster from which data were taken (enclosed by a small black circle) for display and also for statistical analyses.

**Figure 3:**
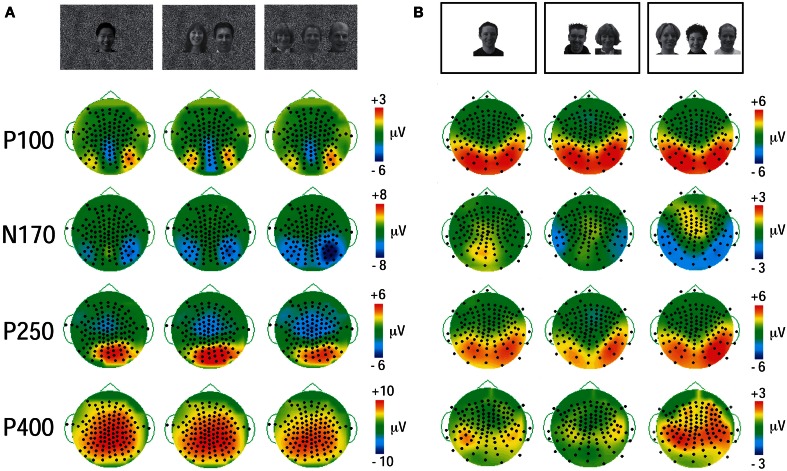
**Topographic voltage maps displaying P100, N170, P250, and P400 ERP activity at their maximal time points. (A)** GBC stimulus set (Experiment 1). Black dots on topographic maps show recording sensor locations. Vertical color calibration bars at the right of each set of maps show microvolt scale—a function of the size of the ERP component. **(B)** LBC stimulus set (Experiment 2). The distribution for components is similar across stimulus sets, but the spatial extent appears to be more focal for the data elicited to equated stimulus set **(A)**.

In both experiments a prominent positive-negative-positive ERP complex consisting of three ERP components (P100, N170, and P250) was elicited to all three viewing conditions (Figure 2) and was maximal at the bilateral temporo-occipital scalp in both experiments (Figure 3). Additionally, a subsequent later positive ERP component (P400) was also seen appeared to be larger to Experiment 1 relative to Experiment 2 (compare each set of waveforms in Figures 2A,B). The amplitudes (Figure 2) and spatial extent (Figure 3) of the ERPs were typically larger in the right hemisphere. The ERP morphology observed here in two experiments was consistent with that elicited in our previous studies (Puce et al., 2000, 2003; Carrick et al., 2007).

We performed identical statistical analyses on the data from each experiment to examine how elicited neural activity was modulated by number of faces. We chose to perform separate analyses given the very large differences in ERP latencies and amplitudes (see above) that were elicited to quite different conditions of visual stimulation. Below, we report on the statistical analysis for the two experiments.

### Experiment 1: preserved global brightness and contrast (GBC)

#### ERP latency differences

**Figure 4:**
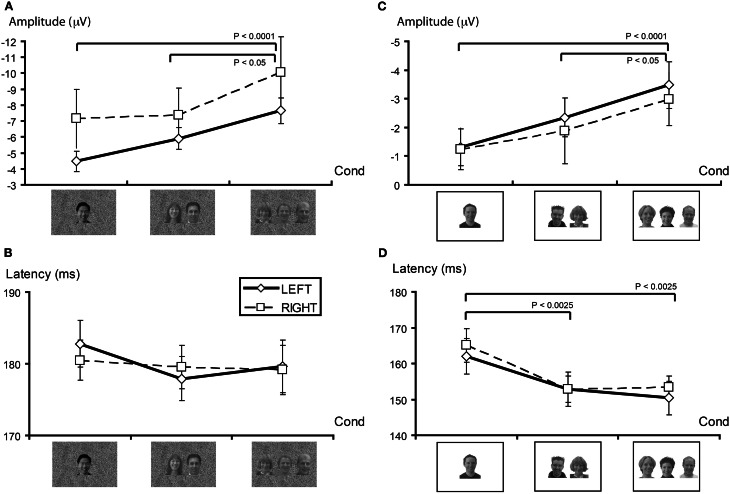
**N170 characteristics as a function of stimulus set. (A)** N170 amplitude (in microvolts) as a function of face number (Cond) for the GBC stimulus set for right and left hemispheres. In both cases, N170 amplitude increases in a graded manner as the number of faces in the display increases. **(B)** N170 latency (in milliseconds) for GBC stimuli did not vary as a function of face number. **(C)** N170 amplitude increases in an approximate linear manner as a function of face number in both hemispheres for LBC stimuli. **(D)** N170 latency decreases significantly as the number of faces in the display increases for LBC stimuli. Legend: solid line plot reflect left hemisphere activity. Broken line plot depicts right hemisphere activity. Significant contrasts between conditions are displayed at the top of each plot.

For both P100 and N170 there were no main effects of condition or hemisphere, or interaction effects (see also Figure 4B for N170 data). Only the later ERPs showed latency differences between conditions. For P250 latency there was a main effect of hemisphere [*F*_(1, 13)_ = 8.70, *P* < 0.025], with significantly longer latencies being observed in the right hemisphere. Similarly, for P400 latency there was also a hemispheric main effect [*F*_(1, 13)_ = 24.19, *P* < 0.001]. However, in contrast to P250, the right hemisphere showed *shorter* latencies for P400. There were no significant interaction effects for the P100, N170, P250, or P400 latency data.

#### ERP amplitude differences

P100 showed no significant main effects across number of faces or recording hemisphere, however, a significant interaction effect was observed [*F*_(2, 26)_ = 5.26, *p* < 0.05]. This interaction effect was produced by significantly larger P100s to the three face condition relative to one and two faces in the left hemisphere (1 vs. 3 *F* = 5.60, *p* < 0.05; 2 vs. 3 *F* = 5.80, *p* < 0.05).

N170 amplitude showed a significant main effect for face condition [*F*_(2, 26)_ = 10.10, *p* < 0.0025; see also Figure 4A] and no main effect for hemisphere or interaction effect. Contrasts indicated N170 amplitude differences between viewing one vs. three faces [*F*_(1, 13)_ = 32.17, *p* < 0.0001], and two vs. three faces [*F*_(1, 13)_ = 4.94, *P* < 0.05], and not for one vs. two faces [*F*_(1, 13)_ = 3.50, ns].

No significant main or interaction effects were observed for P250 or P400 amplitudes.

### Experiment 2: preserved local brightness and contrast (LBC)

#### ERP latency differences

Using stimuli in which local luminance and contrast were not manipulated generated significant main effects of number of faces for both P100 [*F*_(2, 24)_ = 6.06, *p* < 0.01] and N170 [*F*_(2, 24)_ = 13.77, *p* < 0.0001] latencies. The longest latencies were typically observed for the single face display. P100 latency effects were driven by a difference between viewing one vs. three faces [*F*_(1, 12)_ = 8.15, *p* < 0.025], and one vs. two faces [*F*_(1, 12)_ = 7.03, *p* < 0.025], but not for viewing two vs. three faces [*F*_(1, 12)_ = 0.10, ns]. Similarly, for N170 latency there were significant differences between viewing one vs. three faces [*F*_(1,12)_ = 20.23, *p* < 0.0025], and one vs. two faces [*F*_(1, 12)_ = 14.70, *p* < 0.0025], but not two vs. three faces [*F*_(1, 12)_ = 0.54, ns]. These differences are depicted in Figure 4D.

In contrast, P250 and P400 latencies were not affected by viewing different face numbers [P250, *F*_(2, 24)_ = 0.93, ns; P400, *F*_(2, 24)_ = 0.79, ns].

There were no significant main effects of hemisphere or significant interaction effects for P100, N170, P250, or P400 latencies.

#### ERP amplitude differences

Somewhat unexpectedly, no effects of condition were observed for P100 amplitude, nor did it differ across hemispheres, or show an interaction effect.

Similar to the previous experiment, N170 amplitude was again influenced by the number of faces viewed N170 [*F*_(2, 24)_ = 10.80, *p* < 0.005], and this effect was again driven by viewing 3 faces (1 vs. 3, *F* = 33.16, *p* < 0.0001; 2 vs. 3, *F* = 4.94, *p* < 0.05], with no difference being observed between 1 and 2 faces (*F* = 1.816, ns) (see Figure 4C). There were no significant effects of hemisphere, nor was there a significant interaction effect.

Similar to N170, P250 amplitude was found to vary significantly for number of viewed faces [*F*_(2, 24)_ = 3.60, *p* < 0.05]. For P250, contrasts indicated that amplitudes were significantly larger for viewing three faces [1 vs. 3, *F*_(1, 12)_ = 5.55, *p* < 0.05; 2 vs. 3, *F*_(1, 12)_ = 6.77, *p* < 0.05]. Amplitudes to viewing one vs. two faces were not significantly different [*F*_(1, 12)_ = 1.816, ns]. There was a significant effect of hemisphere for P250 amplitude [*F*_(1, 12)_ = 7.93, *P* < 0.025], with P250 amplitude being larger in the right hemisphere overall. No significant interaction effect was observed.

P400 amplitude was not observed to vary as a function of number of faces, or by hemisphere of recording, nor was an interaction effects observed.

### Differences in ERP latencies across experiments

A clear shift in ERP peak latencies was seen when comparing the data across the two experiments (see broken vertical lines linking the respective sets of ERP waveforms in Figure 2). On average for Experiment 1 P100 occurred at around 125 ms when overall brightness and contrast were controlled (GBC), whereas in Experiment 2 an earlier P100 was elicited (peaking at around 104 ms post-stimulus) (LBC). Overall P100 latencies across Experiments 1 and 2 were compared using an unpaired *t*-test and were found to be significantly different across experiments [*t*_(1, 25)_ = 4.57, *P* < 0.0001]. Similarly, N170 peaked at a mean latency of 180 ms for Experiment 1 and 156 ms for Experiment 2 (*t* = 5.26, *P* < 0.0001). P250 peaked at mean latency of 241 ms and 219 ms for Experiments 1 and 2 (*t* = 3.14, *P* < 0.005), respectively. This pattern of latency differences was not as evident in the more broadly distributed P400 (Figures 3A,B bottom row), which tended to be harder to identify in the group averaged data in Experiment 2. P400 peak latencies of 387 ms and 409 ms were calculated using the ERP data of individual participants. These differences were not significant (*t* = 1.24, ns). Overall, there was a systematic difference in ERP peak latencies (P100, N170, and P250) of around 22 ms across the two Experiments, which was most likely driven by the greater overall brightness and contrast of Experiment 2.

Using a similar comparison, mean ERP component amplitudes were also contrasted across experiments. While P100 and P250 differences were not significant (P100 *t* = −0.02, ns; P250 *t* = −0.62, ns), N170 and P400 amplitudes did show significant differences across the two experiments (see also Figure 2) (N170 *t* = 2.95, *P* < 0.01; P400 *t* = 7.87, *P* < 0.0001).

### Results summary

Table 1 summarizes the significant main effects and interactions for all tested ERP components across the two experiments. The effects of stimulus attributes on the P100 and N170 manifested as latency differences in Experiment 2 when the number of faces was varied in a preserved local brightness and contrast environment, and the later component latencies (P250 and P400) appeared to be unaffected by the face number manipulation. When overall brightness and contrast of the stimulus display were controlled P100 and N170 latency effects disappeared, suggesting that these latency differences were caused by differences in low-level stimulus attributes. Interestingly, the later ERP components (P250 and P400) showed latency differences as a function of hemisphere—with significantly larger responses occurring over the right posterior temporal scalp.

**Table 1:** **Summary of statistical analysis of ERP data for both Experiments**.

**Measure**	**Experiment 1: GBC**	**Experiment 2: LBC**
**LATENCY**		
P100	−	+ (Face)
N170	−	+ (Face)
P250	+ (Hemi)	−
P400	+ (Hemi)	−
**AMPLITUDE**		
P100	+ (Face × Hemi)	−
N170	+ (Face)	+ (Face)
P250	−	+ (Hemi), + (Face)
P400	−	−

Significant main effects of number of faces viewed (Face) and hemisphere (Hemi), and interactions (Face X Hemi) are identified for the four different ERP components are tabulated as a function of the preserved global brightness and contrast (GBC) and preserved local brightness and contrast (LBC) stimulus sets. Latency and amplitude data were tested for all ERP components. Legend: +, significant result; −, non-significant result.

Strikingly, N170 amplitude was modulated by the number of faces in the display irrespective of the variation in global or local brightness and contrast (Table 1). Unlike N170, P100 showed a significant interaction effect in the GBC experiment, where the larger number of faces elicited larger P100s in the left hemisphere. Both P250 and P400 amplitudes remained unaffected by the face number manipulation. No effect for P100 or P400 amplitude was observed in the LBC stimulus experiment. P250 showed an interaction effect, with the larger number of faces eliciting the largest amplitudes in the right hemisphere.

There was a significant difference in ERP latencies for P100, N170, and P250 across the two experiments—with latencies being earlier for LBC (Experiment 2) relative to (GBC) Experiment 1.

## Discussion

In this study we performed different types of brightness and contrast manipulations across our two stimulus sets. In the GBC experiment, the overall brightness and contrast of the image was equated. So as to equate the overall brightness and contrast of the stimuli, the local brightness of the face stimuli had to be systematically altered as a function of the increase in face number. In the LBC experiment, overall brightness and contrast of the image varied, whereas local brightness and contrast in the image was preserved. In addition to these brightness and contrast manipulations, other variables that were manipulated were stimulus size (considerably larger visual angles in GBC relative to LBC), as well as stimulus delivery method (GBC: projection on to a white wall from a gray background; LBC presentation on a computer monitor from a black background). Strikingly, despite these many differences in stimulus characteristics, and irrespective of variation in overall or local brightness and contrast, stimulus size, and stimulus presentation from different inter-stimulus backgrounds, and stimulus delivery, N170 amplitude is modulated by the number of viewed faces—with three face displays eliciting the largest responses. The later ERP components showed more complex effects with respect to their amplitudes—when overall luminance and contrast were adjusted to be constant (GBC) no systematic amplitude differences were observed in P250 or P400 amplitudes. In terms of latencies, the early components (P100, N170) showed a significant effect of face number when overall luminance and contrast were allowed to vary (LBC). Comparing the data from the two experiments, a systematic latency difference of around 22 ms was observed, with the ERP data from the LBC experiment where there was a brighter and higher contrast display exhibited the earliest responses. Having said that, differences in stimulus size across the two experiments could also impact ERP latency and amplitude measures, and smaller stimuli should produce smaller ERPs (see McCarthy et al., 1999). In our case, the smaller size for stimuli in the LBC relative to those in the GBC experiment, may well have undercut the latency difference seen between the two experiments: if the stimulus sets had have been the same size, then perhaps the LBC stimuli could have produced even greater differences in latency relative to the GBC stimuli across the two experiments.

### Effects of stimulus brightness, contrast, and size on neural activity

It has long been known that neurophysiological responses to visual stimuli vary their amplitude and latency as a function of stimulus contrast and brightness (Regan, 1972; Halliday et al., 1973; Chiappa, 1983). Diffuse light flashes demonstrated increased amplitudes and decreased latencies to early visual evoked response studies in human subjects (Wicke et al., 1964; Sokol and Riggs, 1971). Later studies using alternating black and white gratings or checkerboards elicited visual evoked responses with decreased latency and increased amplitude to greater brightness and contrast of the visual display (Campbell and Kulikowski, 1972; Regan, 1972). Hence, visual neurophysiologists have always balanced brightness and contrast of visual stimuli, particularly in clinical studies (Chiappa, 1983). This becomes more challenging when using complex stimuli that include faces and objects. It is possible to equate *overall* image brightness and contrast when presenting single static images such as faces and/or objects (Allison et al., 1994, 1999; Bentin et al., 1996). However, this problem becomes significant when dealing with animated visual stimuli or “real-life” activation tasks. In studies using apparent motion stimuli, stimulus frames have been adjusted for these attributes (Carrick et al., 2007), however, this is much harder to accomplish when using stimuli such as movies (Hasson and Malach, 2006; Golland et al., 2007).

With the exception of N170 amplitude, the data from the two experiments indicate that changes in overall and local brightness and contrast not only influence, but can also confound, the response properties of visual evoked responses to higher-order stimuli such as faces. In the LBC data set (Experiment 2), P250 amplitude varied with increasing face number, as a function of the overall brightness and contrast properties of the image, despite local brightness and contrast being preserved. P250 amplitude changes were abolished in the GBC data set, where local brightness and contrast of the faces was systematically manipulated to preserved overall image brightness and contrast (Table 1). The latencies of the later ERP components, P250 and P400, showed latency differences across hemispheres *only* in the experiment where the overall brightness and contrast were controlled, but where the local brightness and contrast (and perhaps discriminability) of the face stimuli was altered. These observations are important, as they contradict a long-held belief that only the earlier ERP components, or those that are associated explicitly with perception, e.g., P100, are susceptible to these low-level stimulus attribute manipulations. Indeed, the systematic latency difference of approximately 22 ms across the two experiments for all ERP components, early and late, indicates the importance of paying attention to these stimulus attributes. This suggests that care be taken when designing paradigms where visual stimulation is complex. Where stimulus brightness and contrast cannot be easily adjusted, perhaps using measures of brightness and contrast as regressors or co-variates in statistical analyses should be considered.

With respect to stimulus issues, our study also had a number of important differences between the two experiments. In Experiment 1, stimuli were projected on a white wall with a video projector with a larger visual angle, whereas in Experiment 2 stimuli were viewed on a computer monitor. It is important to note that despite large differences in stimulus size (much larger stimuli in GBC or Experiment 1 relative to LBC or Experiment 2), significantly shorted ERP latencies were observed in Experiment 2 (LBC). In Experiment 1 inter-stimulus periods consisted of a gray screen upon which the faces embedded in an irregular background were presented, whereas in Experiment 2 a black screen was present in inter-stimulus stimulus periods upon which the stimuli, consisting of the faces embedded in a white rectangle were projected. Differences in how the stimuli appear in their final state, with respect to brightness and contrast, as delivered by the video projector and also the computer monitor are difficult to quantify accurately. We chose to measure the overall brightness and contrast of all stimuli (Figure 1) relative to the images themselves from calculations based on grayscale pixel values. There is always the potential for irregularities in stimulus intensity profiles to occur with video projection or in computer monitors. Having said that, in both experimental manipulations N170 amplitude was found to be sensitive to item number despite large differences in stimulus delivery and size of stimuli, underscoring the robustness of this experimental finding. Indeed, increasing stimulus size has been noted to increase the amplitude and reduce the latency of the N200 elicited to face stimuli (Allison et al., 1999). In our experiment, earlier and larger ERPs were elicited to the *smaller* face stimuli (Experiment 2), whose overall contrast and brightness varied more than that for Experiment 1. We believe that our results reflect the strong effects of overall brightness and contrast on ERPs.

One limitation of our study was that we did not systematically study effects of size and stimulus number in our experiment. Instead we chose to use two very different stimulus sizes and ways of displaying stimuli to subjects so as to look for common neurophysiological effects that might scale with numerosity. Interestingly, for stimuli that consist of arrays of dots it has been reported that ERPs are not affected by the numerosity judgment *per se*, but instead are more likely to be modulated by the visual stimulus size (Gebuis and Reynvoet, 2012, 2013). Unlike for the experiment with dots, *within* each of our experiments, we did demonstrate an effect of numerosity in ERP measures e.g., N170, where visual item size was constant within the experiment. We did also perform an explicit comparison between the data of our two experiments and showed clear differences in ERP latencies and amplitudes that we believe were driven by brightness and contrast—however, it raises the question as to whether these differences would have been even greater if we had have kept our stimulus display sizes the same across the two experiments and varied only brightness and contrast. This is an important line of future investigation for studying neural activity elicited to multiple faces, given that on a daily basis we see other individuals at many different spatial scales, depending on what their respective distance from us is at any given time.

One important, low-level, variable that must also be considered is that of spatial frequency. Faces form a homogeneous class of objects with so–called middle range spatial frequencies e.g., 11 cycles/image, and this property could be a driving factor for eliciting larger N170s to faces relative to objects, as objects typically are much more diverse and have a wider spatial frequency content (Collin et al., 2012). Interestingly, N170 has found to be sensitive to manipulations of spatial frequency for images of faces and objects alike (Collin et al., 2012), however, it is thought that high-spatial frequencies in face stimuli e.g., those greater than 24 cycles/image do not greatly influence the generation of the N170 (Halit et al., 2006). In our study, the increase in face number was also associated with an increase in the amount of middle range spatial frequency information that was presented in the image. An additional set of experiments to investigate the effects of increasing spatial frequency content in images would be needed to ascertain to what extent the increased N170 to face number is influenced directly by this low-level stimulus variable.

### ERP/EEG activity to viewing multiple stimuli from non-face categories

Much focus in the literature has been centered on the N170 that is elicited to faces. Having said that, it has been clearly demonstrated that the N170 ERP is elicited to other stimulus categories such as objects, albeit being significantly smaller relative to that observed to faces (Bentin et al., 1996; Rossion et al., 2003; Guillaume et al., 2009). Intracranial recordings of category-sensitive potentials e.g., N200, thought to be a similar manifestation to the scalp N170 (Rosburg et al., 2010), have clearly shown that category-sensitive ERPs can be elicited to face, face parts, letterstrings, hands, and objects, and N200 amplitude varies as a function of subdural electrode position on the occipitotemporal cortex (Allison et al., 1999; McCarthy et al., 1999; Puce et al., 1999).

There is a growing literature on the neural correlates of numerosity, which has typically used homogeneous visual displays of targets in the form of dots (Gebuis et al., 2010; Ester et al., 2012; Vuokko et al., 2013), usually for the purposes of studying counting and subitizing, or letters or numbers (Gebuis et al., 2010). In displays where targets are presented in a lateralized fashion and are intermixed with non-target distractors, the most predominant ERP component that is observed is a so-called parietal N2pc. N2pc has been found to vary monotonically with increasing target stimulus number for explicit judgments of numerosity (Pagano and Mazza, 2012; Mazza et al., 2013) or for judgments involving subitizing (Ester et al., 2012). As noted earlier, the behavior of N2pc and N1 (analogous to N170 in our study) have been dissociated in ERP studies, where N1 has been found to be modulated by increasing item number when targets are presented without distracters, whereas N2pc will exhibit modulation as a function of numerosity in all types of displays (Mazza et al., 2013). An interesting pattern of results has also emerged as a function of small (e.g., 1–3) vs. large (e.g., 8–24) item number in a passive viewing paradigm. The temporal-occipital N1 (analogous to N170 in our study) varied monotonically for small numbers and not for larger items (Hyde and Spelke, 2009). In this same study a later ERP—the P2 was found to vary as a function of the larger item number. Having said that, some investigators have argued that stimulus size, as determined by the spatial extent of the dot layout, may also be a critical variable when making numerosity judgments (Gebuis and Reynvoet, 2012, 2013). Differences of appearance of targets in foveal, parafoveal and extrafoveal regions of the visual field may also factor into these variations.

An important question that emerges from the data presented in this study is whether the category-sensitive N170, as elicited to other important categories of very complex, but important, stimuli to humans e.g., objects, hands, would also produce graded changes in N170 amplitude for constant brightness, contrast, and stimulus size. Comparing a number of stimulus categories, including scrambled images that preserve the spatial frequency content of the original image, in a series of experiments would be required to successfully resolve this issue.

### Increased arousal and being watched by others

All of us can recall a situation where we might have felt uncomfortable because we were being watched or were the focus of attention of a large group of people. Public speaking is a typical example of where many people report being uncomfortable. Most healthy individuals will report feel some degree of anxiety or discomfort in this situation. What causes the discomfort? It is thought that there is an increase in arousal when being even gazed at even by a single face (Conty et al., 2010b), so that this might be likely to be magnified is the eyes of a group are upon an individual. Associated with this increase in arousal are autonomic nervous system changes such as increased heart and respiration rate, pupillary constriction, “butterflies” in the stomach, a dry mouth, trembling, or shaking (which may also manifest by changes in voice), which can also occur with intense emotions (Stephens et al., 2010).

N170 amplitude increased systematically with increasing face number in *both* experiments. Hence, unrelated to overall or local luminance/contrast changes, an increasing number of faces elicited augmented N170s; however, it could be argued that changes in the spatial layout of the faces might also influence these ERP amplitude changes. The face-sensitive N170 is also typically larger to faces relative to other object categories (Bentin et al., 1996; Eimer and McCarthy, 1999; Taylor et al., 1999; Eimer, 2000; Itier et al., 2006; Rossion and Caharel, 2011), albeit to a lesser extent than to faces (Rossion et al., 2000). It appears that N170 amplitude can be influenced by expertise and training with other object categories (Tanaka and Curran, 2001; Scott et al., 2006).

The underlying mechanisms for the N170 amplitude increase proportional to face number are yet to be elucidated. The data raise a number of interesting, although somewhat unrelated, questions: do more faces simply drive the system more? How does the position of the face grouping in the visual field modulate neural activity? Is it that direct gaze, when experienced from more than one individual, creates a greater arousal effect that results in an augmented N170? Is it that more items in the display drive the system more—an effect that has nothing to do with faces? How do N170s elicited to multiple faces differ to those elicited to the same number of non-face objects in the display? Experiments where these variables are disentangled need to be performed to help elucidate the mechanisms underlying the involuntary and seemingly automatic neurophysiological response that is elicited to the human face.

## Conclusion

In sum, N170 is elicited to a face, and the more faces are present, the larger its amplitude. Importantly, the presence of a complex background or large changes in image brightness, contrast or size do not appear to affect this basic finding. However, given that N170 also shows category-sensitivity to other stimulus categories, a set of experiments further investigating stimulus categories and how these might interact with variables such as spatial frequency would be required to completely resolve this issue.

### Conflict of interest statement

The authors declare that the research was conducted in the absence of any commercial or financial relationships that could be construed as a potential conflict of interest.

## References

[B1] Allison T. Ginter H. McCarthy G. Nobre A. C. Puce A. Luby M. (1994). Face recognition in human extrastriate cortex. J. Neurophysiol. 71, 821–825 8176446 10.1152/jn.1994.71.2.821

[B2] Allison T. Puce A. Spencer D. D. McCarthy G. (1999). Electrophysiological studies of human face perception. I: potentials generated in occipitotemporal cortex by face and non-face stimuli. Cereb. Cortex 9, 415–430 10.1093/cercor/9.5.415 10450888

[B3] Bartels A. Zeki S. (2004). Functional brain mapping during free viewing of natural scenes. Hum. Brain Mapp. 21, 75–85 10.1002/hbm.10153 14755595 PMC6872023

[B4] Bentin S. Allison T. Puce A. Perez A. McCarthy G. (1996). Electrophysiological studies of face perception in humans. J. Cogn. Neurosci. 8, 551–565 10.1162/jocn.1996.8.6.551 20740065 PMC2927138

[B5] Brefczynski-Lewis J. A. Berrebi M. E. McNeely M. E. Prostko A. L. Puce A. (2011). In the blink of an eye: neural responses elicited to viewing the eye blinks of another individual. Front. Hum. Neurosci. 5:68 10.3389/fnhum.2011.00068 21852969 PMC3151614

[B6] Calder A. J. Ewbank M. Passamonti L. (2011). Personality influences the neural responses to viewing facial expressions of emotion. Philos. Trans. R. Soc. Lond. B Biol. Sci. 366, 1684–1701 10.1098/rstb.2010.0362 21536554 PMC3130379

[B7] Campbell F. W. Kulikowski J. J. (1972). The visual evoked potential as a function of contrast of a grating pattern. J. Physiol. 222, 345–356 5033468 10.1113/jphysiol.1972.sp009801 PMC1331385

[B8] Carrick O. K. Thompson J. C. Epling J. A. Puce A. (2007). It's all in the eyes: neural responses to socially significant gaze shifts. Neuroreport 18, 763–766 10.1097/WNR.0b013e3280ebb44b 17471062 PMC2794043

[B9] Chiappa K. H. (1983). Evoked Potentials in Clinical Medicine. New York, NY: Raven Press

[B10] Collin C. A. Therrien M. E. Campbell K. B. Hamm J. P. (2012). Effects of band-pass spatial frequency filtering of face and object images on the amplitude of N170. Perception 41, 717–732 10.1068/p7056 23094460

[B11] Conty L. Gimmig D. Belletier C. George N. Huguet P. (2010a). The cost of being watched: stroop interference increases under concomitant eye contact. Cognition 115, 133–139 10.1016/j.cognition.2009.12.005 20070959

[B12] Conty L. Russo M. Loehr V. Hugueville L. Barbu S. Huguet P. (2010b). The mere perception of eye contact increases arousal during a word-spelling task. Soc. Neurosci. 5, 171–186 10.1080/17470910903227507 19823960

[B13] Conty L. N'Diaye K. Tijus C. George N. (2007). When eye creates the contact! ERP evidence for early dissociation between direct and averted gaze motion processing. Neuropsychologia 45, 3024–3037 10.1016/j.neuropsychologia.2007.05.017 17644145

[B14] Cremers H. R. Demenescu L. R. Aleman A. Renken R. van Tol M. J. van der Wee N. J. (2010). Neuroticism modulates amygdala-prefrontal connectivity in response to negative emotional facial expressions. Neuroimage 49, 963–970 10.1016/j.neuroimage.2009.08.023 19683585

[B15] Demenescu L. R. Kortekaas R. den Boer J. A. Aleman A. (2010). Impaired attribution of emotion to facial expressions in anxiety and major depression. PLoS ONE 5:e15058 10.1371/journal.pone.0015058 21152015 PMC2995734

[B16] Eimer M. (2000). The face-specific N170 component reflects late stages in the structural encoding of faces. Neuroreport 11, 2319–2324 10.1097/00001756-200007140-00050 10923693

[B17] Eimer M. McCarthy R. A. (1999). Prosopagnosia and structural encoding of faces: evidence from event-related potentials. Neuroreport 10, 255–259 10.1097/00001756-199902050-00010 10203318

[B18] Ester E. F. Drew T. Klee D. Vogel E. K. Awh E. (2012). Neural measures reveal a fixed item limit in subitizing. J. Neurosci. 32, 7169–7177 10.1523/JNEUROSCI.1218-12.2012 22623661 PMC3370889

[B19] Gebuis T. Kenemans J. L. de Haan E. H. van der Smagt M. J. (2010). Conflict processing of symbolic and non-symbolic numerosity. Neuropsychologia 48, 394–401 10.1016/j.neuropsychologia.2009.09.027 19804788

[B20] Gebuis T. Reynvoet B. (2012). Continuous visual properties explain neural responses to nonsymbolic number. Psychophysiology 49, 1481–1491 10.1111/j.1469-8986.2012.01461.x 23046492

[B21] Gebuis T. Reynvoet B. (2013). The neural mechanisms underlying passive and active processing of numerosity. Neuroimage 70, 301–307 10.1016/j.neuroimage.2012.12.048 23282277

[B22] George N. Conty L. (2008). Facing the gaze of others. Neurophysiol. Clin. 38, 197–207 10.1016/j.neucli.2008.03.001 18539254

[B23] Golland Y. Bentin S. Gelbard H. Benjamini Y. Heller R. Nir Y. (2007). Extrinsic and intrinsic systems in the posterior cortex of the human brain revealed during natural sensory stimulation. Cereb. Cortex 17, 766–777 10.1093/cercor/bhk030 16699080

[B24] Guillaume C. Guillery-Girard B. Chaby L. Lebreton K. Hugueville L. Eustache F. (2009). The time course of repetition effects for familiar faces and objects: an ERP study. Brain Res. 1248, 149–161 10.1016/j.brainres.2008.10.069 19028466

[B25] Halit H. de Haan M. Schyns P. G. Johnson M. H. (2006). Is high-spatial frequency information used in the early stages of face detection? Brain Res. 1117, 154–161 10.1016/j.brainres.2006.07.059 16999942

[B26] Halliday A. M. McDonald W. I. Mushin J. (1973). Delayed pattern-evoked responses in optic neuritis in relation to visual acuity. Trans. Ophthalmol. Soc. U.K. 93, 315–324 4526453

[B27] Hasson U. Malach R. (2006). Human brain activation during viewing of dynamic natural scenes. Novartis Found. Symp. 270, 203–212 discussion: 212–216, 232–237. 16649716

[B28] Hasson U. Malach R. Heeger D. J. (2010). Reliability of cortical activity during natural stimulation. Trends in Cogn. Sci. 14, 40–48 10.1016/j.tics.2009.10.011 20004608 PMC2818432

[B29] Hasson U. Nir Y. Levy I. Fuhrmann G. Malach R. (2004). Intersubject synchronization of cortical activity during natural vision. Science 303, 1634–1640 10.1126/science.1089506 15016991

[B30] Haxby J. V. Hoffman E. A. Gobbini M. I. (2000). The distributed human neural system for face perception. Trends Cogn. Sci. 4, 223–233 10.1016/S1364-6613(00)01482-0 10827445

[B31] Hyde D. C. Spelke E. S. (2009). All numbers are not equal: an electrophysiological investigation of small and large number representations. J. Cogn. Neurosci. 21, 1039–1053 10.1162/jocn.2009.21090 18752403 PMC2735795

[B32] Itier R. J. Alain C. Kovacevic N. McIntosh A. R. (2007). Explicit versus implicit gaze processing assessed by ERPs. Brain Res. 1177, 79–89 10.1016/j.brainres.2007.07.094 17916340

[B33] Itier R. J. Batty M. (2009). Neural bases of eye and gaze processing: the core of social cognition. Neurosci. Biobehav. Rev. 33, 843–863 10.1016/j.neubiorev.2009.02.004 19428496 PMC3925117

[B34] Itier R. J. Latinus M. Taylor M. J. (2006). Face, eye and object early processing: what is the face specificity? Neuroimage 29, 667–676 10.1016/j.neuroimage.2005.07.041 16169749

[B35] Kuefner D. de Heering A. Jacques C. Palmero-Soler E. Rossion B. (2010). Early visually evoked electrophysiological responses over the human brain (P1, N170) show stable patterns of face-sensitivity from 4 years to adulthood. Front. Hum. Neurosci. 3:67 10.3389/neuro.09.067.2009 20130759 PMC2805434

[B36] Mazza V. Pagano S. Caramazza A. (2013). Multiple object individuation and exact enumeration. J. Cogn. Neurosci. 25, 697–705 10.1162/jocn_a_00349 23249353

[B37] McCarthy G. Puce A. Belger A. Allison T. (1999). Electrophysiological studies of human face perception. II: response properties of face-specific potentials generated in occipitotemporal cortex. Cereb. Cortex 9, 431–444 10.1093/cercor/9.5.431 10450889

[B38] Meeren H. K. Hadjikhani N. Ahlfors S. P. Hamalainen M. S. de Gelder B. (2008). Early category-specific cortical activation revealed by visual stimulus inversion. PLoS ONE 3:e3503 10.1371/journal.pone.0003503 18946504 PMC2566817

[B39] Meshulam M. Ramot M. Harel M. Kipervasser S. Andelman F. Neufeld M. Y. (2013). Selectivity of audio-visual ECoG responses revealed under naturalistic stimuli in the human cortex. J. Neurophysiol. [Epub ahead of print]. 10.1152/jn.00474.2012 23407355

[B40] Pagano S. Mazza V. (2012). Individuation of multiple targets during visual enumeration: new insights from electrophysiology. Neuropsychologia 50, 754–761 10.1016/j.neuropsychologia.2012.01.009 22266261

[B41] Privman E. Nir Y. Kramer U. Kipervasser S. Andelman F. Neufeld M. Y. (2007). Enhanced category tuning revealed by intracranial electroencephalograms in high-order human visual areas. J. Neurosci. 27, 6234–6242 10.1523/JNEUROSCI.4627-06.2007 17553996 PMC6672161

[B42] Puce A. Allison T. McCarthy G. (1999). Electrophysiological studies of human face perception. III: effects of top-down processing on face-specific potentials. Cereb. Cortex 9, 445–458 10.1093/cercor/9.5.445 10450890

[B43] Puce A. Smith A. Allison T. (2000). ERPs evoked by viewing facial movements. Cogn. Neuropsychol. 17, 221–239 10.1080/026432900380580 20945181

[B44] Puce A. Syngeniotis A. Thompson J. C. Abbott D. F. Wheaton K. J. Castiello U. (2003). The human temporal lobe integrates facial form and motion: evidence from fMRI and ERP studies. Neuroimage 19, 861–869 10.1016/S1053-8119(03)00189-7 12880814

[B45] Regan D. (1972). Evoked Potentials in Psychology, Sensory Physiology and Clinical Medicine. London: Chapman and Hall 10.1007/978-94-011-6890-8

[B46] Rosburg T. Ludowig E. Dumpelmann M. Alba-Ferrara L. Urbach H. Elger C. E. (2010). The effect of face inversion on intracranial and scalp recordings of event-related potentials. Psychophysiology 47, 147–157 10.1111/j.1469-8986.2009.00881.x 19761525

[B47] Rossion B. Caharel S. (2011). ERP evidence for the speed of face categorization in the human brain: disentangling the contribution of low-level visual cues from face perception. Vis. Res. 51, 1297–1311 10.1016/j.visres.2011.04.003 21549144

[B48] Rossion B. Gauthier I. Tarr M. J. Despland P. Bruyer R. Linotte S. (2000). The N170 occipito-temporal component is delayed and enhanced to inverted faces but not to inverted objects: an electrophysiological account of face-specific processes in the human brain. Neuroreport 11, 69–74 10.1097/00001756-200001170-00014 10683832

[B49] Rossion B. Joyce C. A. Cottrell G. W. Tarr M. J. (2003). Early lateralization and orientation tuning for face, word, and object processing in the visual cortex. Neuroimage 20, 1609–1624 10.1016/j.neuroimage.2003.07.010 14642472

[B50] Scott L. S. Tanaka J. W. Sheinberg D. L. Curran T. (2006). A reevaluation of the electrophysiological correlates of expert object processing. J. Cogn. Neurosci. 18, 1453–1465 10.1162/jocn.2006.18.9.1453 16989547

[B51] Senkowski D. Saint-Amour D. Kelly S. P. Foxe J. J. (2007). Multisensory processing of naturalistic objects in motion: a high-density electrical mapping and source estimation study. Neuroimage 36, 877–888 10.1016/j.neuroimage.2007.01.053 17481922

[B52] Sokol S. Riggs L. A. (1971). Electrical and psychophysical responses of the human visual system to periodic variation of luminance. Invest. Ophthalmol. 10, 171–180 5548199

[B53] Stephens C. L. Christie I. C. Friedman B. H. (2010). Autonomic specificity of basic emotions: evidence from pattern classification and cluster analysis. Biol. Psychol. 84, 463–473 10.1016/j.biopsycho.2010.03.014 20338217

[B54] Tanaka J. W. Curran T. (2001). A neural basis for expert object recognition. Psychol. Sci. 12, 43–47 10.1111/1467-9280.00308 11294227

[B55] Taylor M. J. Batty M. Itier R. J. (2004). The faces of development: a review of early face processing over childhood. J. Cogn. Neurosci. 16, 1426–1442 10.1162/0898929042304732 15509388

[B56] Taylor M. J. McCarthy G. Saliba E. Degiovanni E. (1999). ERP evidence of developmental changes in processing of faces. Clin. Neurophysiol. 110, 910–915 10.1016/S1388-2457(99)00006-1 10400205

[B57] Towler J. Eimer M. (2012). Electrophysiological studies of face processing in developmental prosopagnosia: neuropsychological and neurodevelopmental perspectives. Cogn. Neuropsychol. 29, 503–529 10.1080/02643294.2012.716757 23066851

[B58] Tsao D. Y. Livingstone M. S. (2008). Mechanisms of face perception. Ann. Rev. Neurosci. 31, 411–437 10.1146/annurev.neuro.30.051606.094238 18558862 PMC2629401

[B59] Vuokko E. Niemivirta M. Helenius P. (2013). Cortical activation patterns during subitizing and counting. Brain Res. 1497, 40–52 10.1016/j.brainres.2012.12.019 23268353

[B60] Whittingstall K. Bartels A. Singh V. Kwon S. Logothetis N. K. (2010). Integration of EEG source imaging and fMRI during continuous viewing of natural movies. Magn. Reson. Imaging 28, 1135–1142 10.1016/j.mri.2010.03.042 20579829

[B61] Wicke J. D. Donchin E. Lindsley D. B. (1964). Visual evoked potentials as a function of flash luminance and duration. Science 146, 83–85 10.1126/science.146.3640.83 14173038

